# 846. Venovenous ECMO for Burn Patients with Acute Hypoxemic Respiratory Failure: Single Center Case Series

**DOI:** 10.1093/jbcr/irag033.316

**Published:** 2026-04-06

**Authors:** Jude Jaraki, Jan V Stevens, Felix Shun, David Springstead, Adam Olszewski, Yusuke Terasaki, Heather Dolman, Michael T White, Andrew Isaacson, Steven Tennenberg

**Affiliations:** Wayne State University School of Medicine, Detroit, MI; Wayne State University School of Medicine, Grosse Pointe Park, MI; Wayne State University School of Medicine, Detroit, MI; Wayne State University School of Medicine, Detroit, MI; Wayne State University School of Medicine, Detroit, MI; Wayne State University School of Medicine, Detroit, MI; Detroit Receiving Hospital Burn Center, Detroit, MI; Detroit Receiving Hospital Burn Center, Detroit, MI; Wayne State University School of Medicine, Detroit, MI; Wayne State University School of Medicine, Detroit, MI

## Abstract

**Introduction:**

Hypoxemic respiratory failure is a common cause of mortality in burn patients. Complications of burns including inhalation injury, pulmonary edema, sepsis, and pneumonia increase patients’ risk of developing acute respiratory distress syndrome (ARDS). Current ARDS management includes lung-protective mechanical ventilation, prone positioning, promoting negative fluid balance, and glucocorticoids. Adjuncts of inhaled vasodilators and neuromuscular blockade show less-defined mortality benefit. In cases of severe ARDS with refractory hypoxemia, venovenous extracorporeal membrane oxygenation (VV-ECMO) can provide pulmonary support as the patient’s disease course resolves. Data on ECMO’s benefit in this patient population remain limited.

**Methods:**

This IRB-approved retrospective case series identified adult patients from our ABA-verified burn center who underwent VV-ECMO at our ECMO-capable sister tertiary care hospital between July 2020 and December 2024. We included patients with acute burn or inhalation injuries who developed severe ARDS or life-threatening hypoxemia refractory to conventional therapy.

**Results:**

We identified 10 patients who underwent VV-ECMO with a mean age of 46.1 ± 15.0 (mean ± standard deviation) years. Six patients had flame burns, 3 had isolated inhalation injury, and 1 had an electrical injury. The 7 patients with cutaneous burns had a mean total burn surface area of 16.0 ± 13.0%, ranging from 0.3% to 35%. The mean PaO2/FiO2 ratio immediately prior to ECMO was 60.2 ± 18.8. Mean time to ECMO was 7.3 ± 10.1 days from admission and mean ECMO duration was 21.4 ± 19.8 days. Two patients were extubated while on ECMO. During ECMO, 8 patients required vasopressors, 5 underwent tracheostomy, 5 required transfusion for major bleeding, and 2 required renal replacement therapy. Nine patients (90%) survived to discharge, with 6 discharged home, 1 discharged to a long-term acute care facility, 1 discharged to an inpatient rehabilitation unit, and 1 discharged to an inpatient psychiatric ward.

**Conclusions:**

In this small case series of burn-associated severe ARDS and hypoxemic respiratory failure, VV-ECMO rescue therapy resulted in a low mortality rate (10%). These findings demonstrate that ECMO can be a life-saving component of a multimodal critical care approach to burns with severe ARDS. Further studies are needed to define ECMO’s mortality benefit in this patient population.

**Applicability of Research to Practice:**

Burn care providers should consider VV-ECMO as an adjunct therapy for acute hypoxemic respiratory failure and severe ARDS that fails to respond to traditional evidence-based therapies. As ECMO utilization in burn care grows, providers should be cognizant of their centers’ ECMO capabilities and seek early consultation with ECMO specialists.

**Funding for the study:**

N/A.

